# Correlation Between Baseline Serum Testosterone Levels and Cognitive Gain Following Combined Oral Contraceptive Treatment in Patients With Polycystic Ovary Syndrome

**DOI:** 10.7759/cureus.108574

**Published:** 2026-05-09

**Authors:** Saloni Kumari, Sheeba Marwah, Soumen Manna, Himani Ahluwalia

**Affiliations:** 1 Physiology, Vardhman Mahavir Medical College and Safdarjung Hospital, New Delhi, IND; 2 Obstetrics and Gynaecology, Vardhman Mahavir Medical College and Safdarjung Hospital, New Delhi, IND

**Keywords:** cognition function, combined oral contraceptives, female hyperandrogenism, polycystic ovary syndrome (pcos), total testosterone

## Abstract

Background

Combined oral contraceptives (COCs) are the cornerstone therapy for polycystic ovary syndrome (PCOS). While COCs have been shown to improve cognitive performance, the relationship between these gains and hormonal profiles, especially testosterone, remains unclear. This study investigated whether baseline testosterone levels correlate with cognitive changes after COC treatment.

Methods

This quasi-experimental study evaluated 35 nulliparous women (aged 18-35 years) with PCOS. Baseline total serum testosterone levels were measured before starting a three-cycle course of COCs (levonorgestrel and ethinyl estradiol). Cognitive function, working memory (digit span test or DST), attention (continuous performance test or CPT), and executive function (trail making test or TMT) were assessed at baseline and post-treatment. Spearman’s correlation was used to analyze the relationship between baseline serum testosterone levels and individual cognitive change scores (Δ values).

Results

The cohort had a mean age of 21.1 years (BMI of 24.1 kg/m^2^) and a mean baseline testosterone level of 33.6 ng/dL. Analysis revealed no statistically significant correlation between baseline testosterone levels and changes in any cognitive parameters (DST, CPT, TMT), except for a statistical correlation between basal serum testosterone and change in mean RT of the hit rate in continuous performance test, identical pairs (CPT-IP) (p=0.04, r=0.34).

Conclusions

The study did not provide sufficient evidence to conclude that baseline testosterone levels have any predictive or associative relationship with cognitive improvement following three months of COC therapy in women with PCOS. These findings suggest that cognitive gains in this population are likely influenced by factors beyond the baseline total testosterone concentrations.

## Introduction

Polycystic ovary syndrome (PCOS) is a prevalent endocrine disorder that affects many women of reproductive age and is characterized by hyperandrogenism, menstrual irregularities, and ovulatory dysfunction [1,2]. The management of PCOS often necessitates a multifaceted approach, with oral contraceptives (OCs) serving as the cornerstone therapy targeting key features, including irregular menstrual cycles and clinical hyperandrogenism, while simultaneously providing endometrial protection [3]. Combined oral contraceptives (COCs), which comprise estrogen and progestin, have demonstrated efficacy in regulating menstrual cycles, decreasing androgen levels, and ameliorating conditions, such as hirsutism and acne, commonly associated with PCOS [4]. A randomized clinical trial by Alpanes et al. demonstrated that the combination of COCs and spironolactone significantly reduced serum androgen levels and provided symptomatic relief in hirsutism and menstrual irregularities [5].

Women with PCOS exhibit a consistent pattern of cognitive impairment, particularly affecting attention, memory, executive functioning, and information processing speed [6]. Recent longitudinal studies have shown that women with PCOS demonstrate lower cognitive performance and reduced white matter integrity than their non-PCOS counterparts, with midlife women scoring approximately 11% lower on attention, verbal learning, and memory tasks [7]. They also found that a lower cognitive score was associated with high androgen levels, especially free testosterone levels, in women with PCOS [7]. Recently, Redkar et al. reported that women with PCOS had more errors and slower reaction times in focused attention tasks compared to a control group, possibly due to impaired cognitive control and working memory, with neuroanatomical and neurotransmitter alterations implicated [8]. However, they did not analyze the relationship between hormonal profiles and cognitive scores in their study [8].

Schattmann and Sherwin suggested that elevated free testosterone levels in women with PCOS are associated with poorer performance on female-favoring cognitive tasks, while not enhancing performance on male-favored tasks [9]. In another study, hormonal treatment reduced free testosterone levels in women with PCOS, but did not significantly improve cognitive performance, except for verbal fluency, suggesting that cognitive deficits may not be easily reversed by such treatment [10]. However, Soleman et al. showed that anti-androgenic treatment appeared to improve cognitive functioning in women with PCOS compared to that in the control group [11].

The effects of oral contraceptive therapy on cognitive domains in women are limited, and the findings remain mixed, with improvements in cognitive function sometimes reflecting practice effects rather than true pharmacological benefits, and the degree of cognitive response may vary depending on the PCOS phenotype and the individual’s metabolic status.

In our preliminary study on a small group of women with PCOS, we found that three months of COCs treatment improved cognitive performance in the domains of working memory, attention, and executive function [12]. However, we could not support these improvements with hormonal data. Therefore, this study aimed to correlate baseline testosterone levels with changes in cognitive parameters after 3 months of COC treatment in patients with PCOS. Given the established link between hyperandrogenism and altered cognitive processing in PCOS, the current study hypothesized that baseline testosterone would serve as a physiological predictor of cognitive gain in patients with PCOS.

## Materials and methods

A quasi-experimental study was conducted on 35 nulliparous women aged 18-35 years, diagnosed with polycystic ovary syndrome (PCOS) based on the 2003 Rotterdam criteria [13], and attending the Obstetrics and Gynecology outpatient department. The inclusion and exclusion criteria and sample size calculation are described in our previous article by Kumari et al. [12].

Clinical assessment and data collection

The protocol was approved by the Institutional Ethical Committee of Vardhman Mahavir Medical College and Safdarjung Hospital, New Delhi (No: IEC/VMMC/SJH/THESIS/2023/CC-257) before starting any work related to the study, and written informed consent was obtained from each participant in accordance with the principles of the Declaration of Helsinki.

Informed written consent was obtained from all participants in English or Hindi before the study. A detailed history, including age, educational status, and medical history, was recorded using a specially designed proforma. Anthropometric measurements (height, weight, and calculated BMI) were taken with the participants in light clothing and bare feet.

Baseline total serum testosterone levels were obtained from all participants before the initiation of COC treatment. Biochemical estimation of serum testosterone was performed in all patients with PCOS as per the routine treatment protocol of the outpatient department of Obstetrics and Gynecology at our hospital. Total testosterone was estimated using a fully automated chemiluminescent immunoassay on the AutoLumo A2000 Plus system (Autobio, Zhengzhou, China).

Computer-based cognitive function testing

Participants were given sufficient rest and advised to refrain from caffeine consumption on the day of the assessment. The participants were seated comfortably in a chair, and they were made familiar with the apparatus and the procedure to alleviate any fear or apprehension.

Computer-based cognition tests were administered in the morning using Inquisit 6 software (Millisecond, USA, https://www.millisecond.com/). The second session of cognitive function testing was performed after three cycles of ongoing COC treatment containing levonorgestrel and ethinyl estradiol for each participant. All cognitive function assessments were performed between days 1 and 5 of the menstrual cycle. Details of the COC treatment are provided in our previous study [12].

Cognitive Function Testing Covering Three Domains

(i) Working memory (WM) was assessed using the verbal/auditory and visuospatial forward digit span test (fDST). Both tasks required participants to recall digit sequences in the order presented by selecting the digits on a computer screen. The total time for each part was four minutes. The measurements included the two-error maximal length (TEML), two-error total trials (TETT), maximal forward digit span (ML), and mean span (MS) tasks.

(ii) Sustained and selective attention were assessed using the continuous performance test, identical pairs (CPT-IP). Participants pressed a key when a four-digit number was repeated (Go trials) and waited for non-repeating stimuli (NoGo trials). The task took 3 min. The measurements included the mean reaction time (RT) hit rate, mean RT false alarm rate (RTFA), mean RT random errors (RTRE), Z score of hit rate (ZHR), false alarm rate (ZFA), and d prime (parametric sensitivity).

(iii) Executive functioning and task switching were assessed using the trail making test (TMT). The participants used a mouse to connect a predetermined sequence of numbers and letters as quickly and accurately as possible. The task took approximately 5 min to complete. The recorded measurements were trail 1 errors (T1E) and time (T1T), trail 2 errors (T2E) and time (T2T), combined trail errors (CTE), and combined trail time (CTT).

Data analysis

All outcomes of cognitive function were digitally recorded, and the individual change score (Δ value) for each parameter was calculated by subtracting the pre-treatment measurement from the post-treatment measurement using Microsoft Excel (Microsoft Corp., Redmond, WA, USA). Subsequently, all Δ values were imported into GraphPad Prism v. 9 for definitive statistical analysis. The normality of the data distribution was assessed using the Shapiro-Wilk test. Spearman’s correlation coefficients were calculated to analyse the relationship between all Δ values of the cognitive parameters and serum testosterone levels.

## Results

The mean age of the study population was 21.1 years, height 155 cm, weight 57.6 kg, and BMI 24.1 (Table 1). The average age at menarche was 12.5 years, with a mean menstrual cycle length of 47.2 days and a mean duration of menstrual bleeding of 4.9 days per cycle.

**Table 1:** Distribution of demographic data of the study population IQR: interquartile range

	Mean ± SD	Median (IQR)
Age (in years)	21.1±4.3	19 (18–24)
Height (in cm)	155.5±10.3	154 (152–161)
Weight (in kg)	57.6±10.5	55 (49–65)
BMI (in kg/m^2^)	24.1±5.2	23 (20.3–25.9)
Length of each menstrual cycle (in days)	47.2±19.4	45 (30–55)
Duration of bleeding in each cycle (in days)	4.9±1.1	5 (3–7)
Baseline Total Serum Testosterone (ng/dL)	33.6±15.3	28.34 (25.2–38.1)

The correlations between baseline serum testosterone levels and changes in auditory and visual digit span test parameters (Δ values) are presented in Table 2. However, no statistically significant monotonic correlation was observed between the two variables.

**Table 2:** Correlation between changes of auditory and visual digit span test parameters after COC treatment and baseline serum testosterone levels of patients A non-parametric test, Spearman’s correlation, was used. The mean value of different auditory digit span test parameters and serum testosterone level has been used for the correlation study. r = correlation coefficient; p-value = 2-tailed significance; COC: combined oral contraceptive

		Baseline Serum Testosterone	
		r	p-Value
Change in TEML	Auditory	-0.1066	0.5421
	Visual	-0.2621	0.1283
Change in TETT	Auditory	-0.1161	0.5067
	Visual	-0.2106	0.2247
Change in ML	Auditory	-0.0095	0.9567
	Visual	-0.3226	0.0587
Change in MS	Auditory	-0.06282	0.7200
	Visual	-0.3316	0.0517

**Figure 1:**
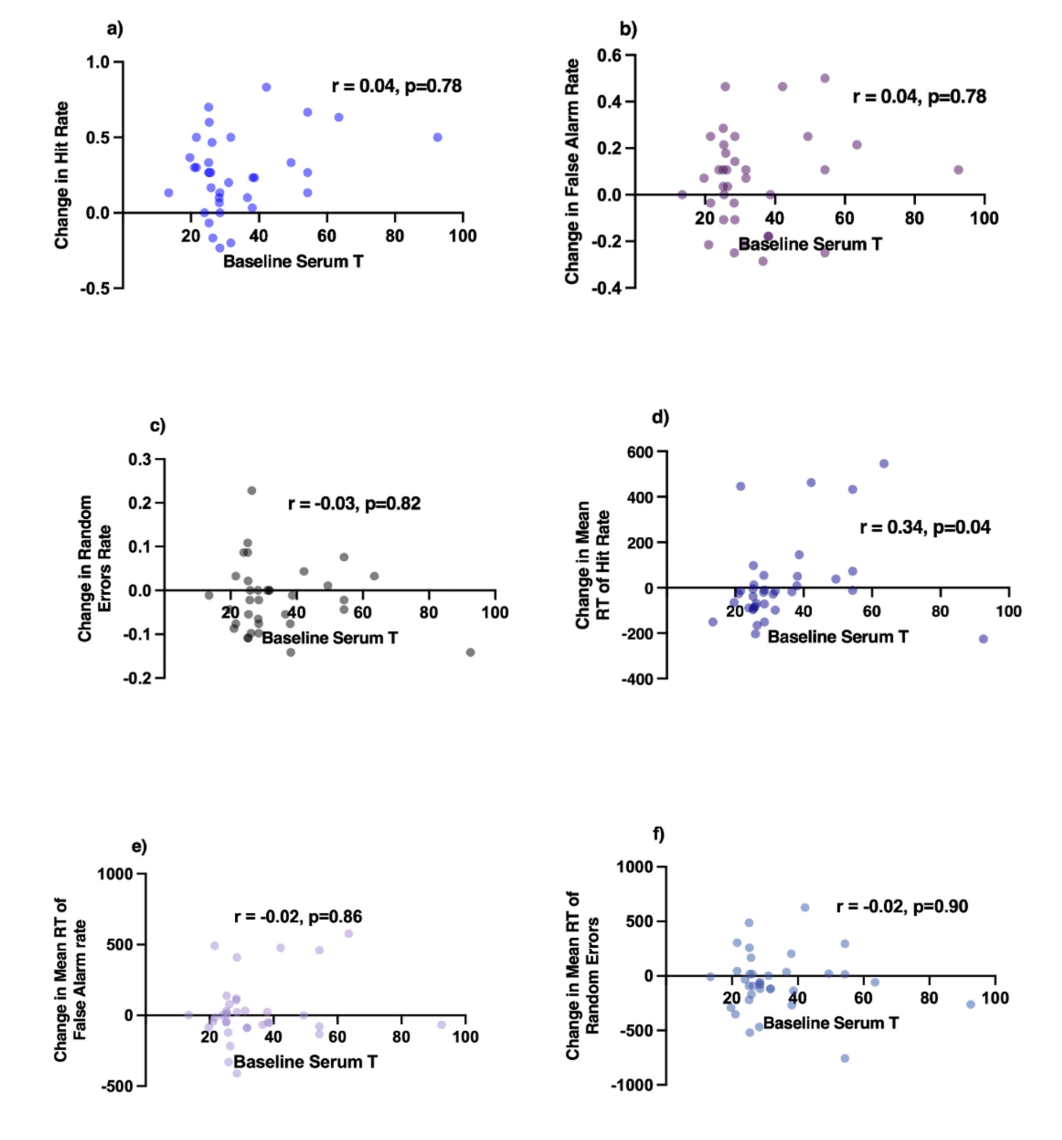
(a to f) Correlation between changes in CPT-IP parameters (Δ values) and baseline serum testosterone levels. CPT-IP: continuous performance test, identical pairs

**Figure 2:**
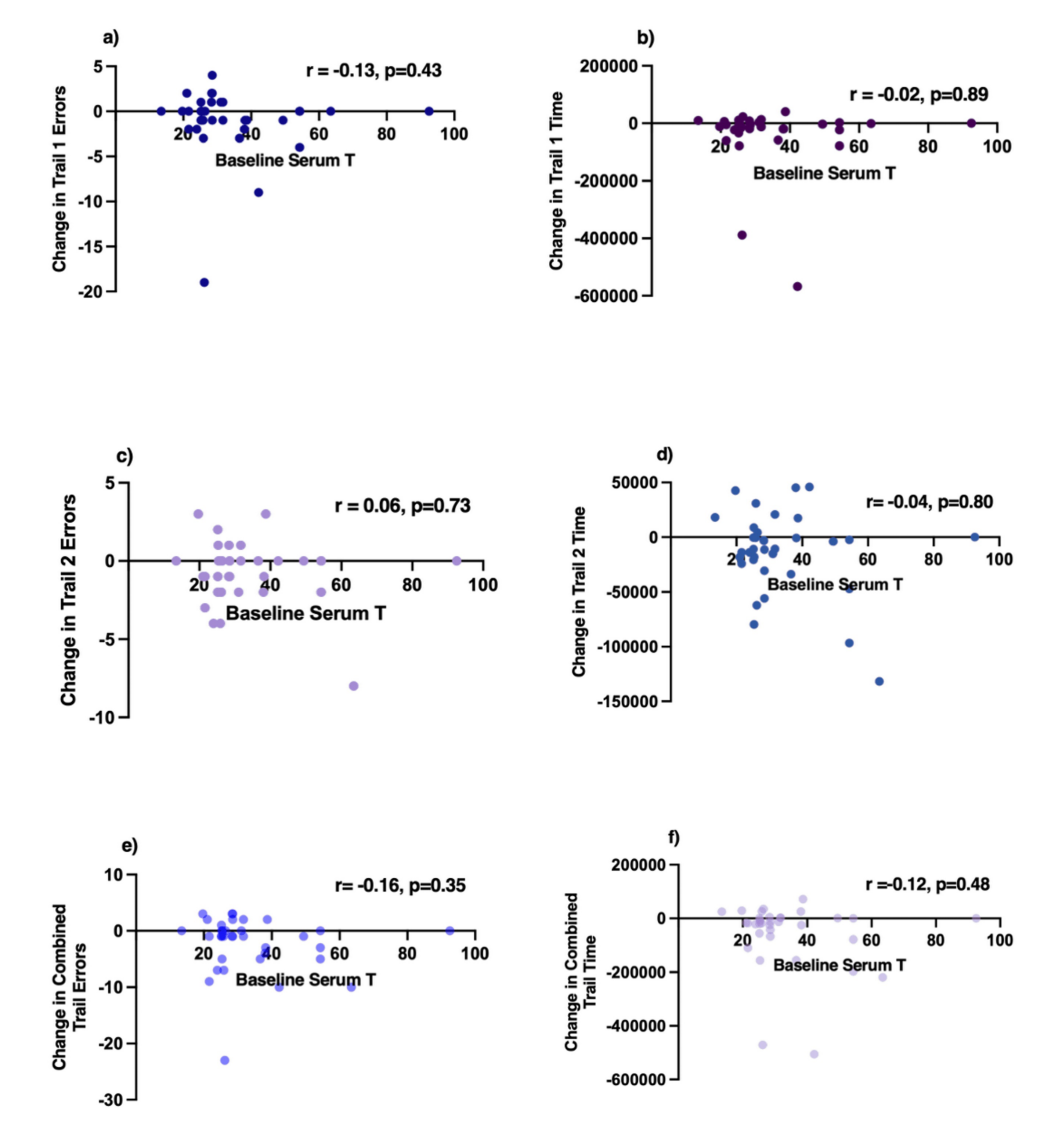
(a to f) Correlation between changes in TMT parameters (Δ values) and baseline serum testosterone levels. TMT: trail making test

The correlation between baseline serum testosterone and gain in CPT-IP parameters (Δ values) and gain in TMT parameters (Δ values) are presented in Figure 1 and Figure 2, respectively. Basal serum testosterone level showed a statistically significant correlation (p=0.04, r=0.34) with the change in the mean RT of the hit rate in the CPT-IP (Figure 1). For all other parameters of the CPT-IP and TMT, no statistically significant monotonic correlations were observed.

## Discussion

The primary objective of this study was to determine whether baseline serum testosterone levels serve as a predictor of the degree of cognitive performance change following three months of COC treatment in patients with PCOS. This study utilized changes in the auditory and visual fDST, CPT-IP, and TMT scores as indicators of cognitive improvement. Contrary to the hypothesis that higher baseline hyperandrogenism would predict a greater benefit from androgen-lowering therapy, the analysis revealed no statistically significant correlation between the mean baseline serum testosterone level and the change (improvement) in any of the cognitive parameters examined.

This study focused on a population with a mean age of 21.1 years and a mean BMI of 24.1 kg/m², indicating a younger, overweight study population.​ The results demonstrated low correlation coefficients (r) between baseline serum testosterone levels and changes in cognitive test scores after COC therapy. However, none of these reached statistical significance, except for a statistical correlation between basal serum testosterone and change in mean RT of the hit rate in CPT-IP (p=0.04, r=0.34). However, given the high variability in hormonal data and the limited sample size, the observed correlation (p=0.04) should be interpreted as nominal and exploratory, as it may be influenced by individual outliers within the small cohort. The lack of a significant correlation challenges the hypothesis that androgen status may modulate cognitive outcomes, at least in the context of COC-mediated hormonal regulation.

Previous studies have suggested that hyperandrogenism in patients with PCOS may affect cognitive domains, particularly those involving executive functioning and memory [9,14,15]. However, the current data indicate that changes in fDST, CPT-IT, and TMT scores after three months of COCs therapy were not substantially attributable to the initial testosterone concentration. These findings that baseline testosterone was not significantly correlated with cognitive improvement suggest that the mechanisms underpinning the neurocognitive benefits of COCs in PCOS are more complex than a simple dose-response relationship with pre-treatment androgen levels. This outcome is consistent with some prior studies, indicating that while pharmacologic manipulation of free testosterone significantly reduces androgen levels in patients with PCOS, the impact on broad cognitive function is often minimal, except possibly for verbal fluency [10].

COCs act primarily by suppressing ovarian androgen synthesis and significantly increasing sex hormone-binding globulin (SHBG), which drastically lowers the amount of free, biologically active testosterone [4,16,17]. The cognitive improvement observed in patients with PCOS following COC treatment may thus be driven not by the initial testosterone concentration, but rather by the dramatic reduction in bioavailable androgens (free testosterone) or by other concurrent hormonal and metabolic changes induced by COCs. This might be due to a threshold effect, where achieving a certain low level of free androgen, irrespective of the starting point, is sufficient for cognitive benefit, rather than a linear predictive relationship based on baseline total testosterone.

While the hyperandrogenism hypothesis remains central to the pathophysiology of PCOS, mounting evidence suggests that metabolic dysfunction, particularly insulin resistance (IR), may be a more dominant mediator of cognitive impairment in this population [18,19]. IR is highly prevalent in PCOS and impairs frontal lobe metabolism, cognitive flexibility, and attentional capacity [20]. Since COCs can have mixed effects on insulin sensitivity, the observed cognitive changes may reflect a balance between the positive effects of androgen reduction and the potential negative effects on glucose metabolism, masking any correlation with baseline testosterone [18,21].

From a clinical perspective, these results provide useful insights. Since testosterone levels did not strongly influence cognitive enhancement after COC use, clinicians may focus on other factors, such as psychosocial interventions, metabolic health, or mood disorders, when aiming to improve cognitive functioning in patients with PCOS. COC therapy remains an effective approach for regulating hormonal and menstrual imbalances in PCOS, but its cognitive benefits appear to be independent of the pre-treatment testosterone status in this cohort.

Limitations

The sample size of the study was small and mainly focused on testosterone levels and cognitive function. In addition, the study lacked a control group. Further research should explore whether other cognitive functions, such as spatial reasoning or verbal fluency, show a stronger correlation with androgen levels and SHBG. Moreover, longitudinal designs involving long-term COC exposure and larger populations could clarify subtle associations that may have been missed in the current design. Specifically, follow-up investigations should employ free androgen index (FAI) or free testosterone measurements, given their biological relevance, and include markers of insulin resistance (HOMA-IR) and inflammation in multivariate models to fully elucidate the complex drivers of neurocognitive response to COC therapy in PCOS.

## Conclusions

The data do not provide sufficient evidence to conclude that baseline testosterone levels have any predictive or associative relationship with the changes in cognitive parameters over time after COCs treatment in women with PCOS. This suggests that the neurocognitive benefits of COCs may be mediated by broader mechanisms, such as the stabilization of the hypothalamic-pituitary-ovarian axis or improvements in metabolic factors, rather than by androgen reduction alone. Although COCs remain an effective treatment for hyperandrogenism and are associated with cognitive improvement, future research should focus on alternative or combined predictors of cognitive improvement. 
